# Non-tuberculous mycobacterial disease associated with *Mycobacterium montefiorense* in salamanders

**DOI:** 10.3389/fvets.2023.1248288

**Published:** 2023-10-26

**Authors:** Takeshi Komine, Hyogo Ihara, Mari Inohana, Jennifer Caroline Kwok, Akane Shimizu, Tsumugi Terasawa, Ayaka Miyazaki, Saralee Srivorakul, Hajime Iwao, Sachiko Harada, Mitsunori Yoshida, Yoshihiko Hoshino, Osamu Kurata, Hanako Fukano, Shinpei Wada

**Affiliations:** ^1^Laboratory of Aquatic Medicine, School of Veterinary Medicine, Nippon Veterinary and Life Science University, Musashino, Tokyo, Japan; ^2^Department of Mycobacteriology, Leprosy Research Center, National Institute of Infectious Diseases, Higashimurayama, Tokyo, Japan; ^3^Retinal Disease Studies Facility, School of Veterinary Medicine, University of Pennsylvania, Philadelphia, PA, United States; ^4^Center of Veterinary Diagnosis and Technology Transfer, Faculty of Veterinary Medicine, Chiang Mai University, Chiang Mai, Thailand; ^5^Niigata City Aquarium, Niigata, Japan

**Keywords:** infection, pathology, whole-genome sequencing, pathogenesis, phylogeny, Mycobacterium montefiorense, salamander

## Abstract

**Introduction:**

*Mycobacterium montefiorense* is one of the causes of non-tuberculous mycobacterial infections in moray eels and salamanders. Although *M. montefiorense* infection could be a threat to salamanders, little information is available regarding this pathogen and associated infection. This study aimed to provide fundamental information regarding *M. montefiorense* and its infection in salamanders.

**Methods:**

Nine *M. montefiorense* strains isolated from three species of salamanders, namely, Japanese black salamander (*Hynobius nigrescens*), Hakuba salamander (*H. hidamontanus*), and Tohoku hynobiid salamander (*H. lichenatus*), between 2010 and 2018, were characterized based on phenotypic and genetic examination. We also pathologically observed salamanders infected with the *M. montefiorense* strains, including Hakuba salamanders and Tohoku hynobiid salamanders.

**Results:**

The microbiological and chemical characteristics of the *M. montefiorense* salamander and an eel strain (reference strain) matched. Susceptibility testing for antimicrobials suggested that clarithromycin may be effective. Regarding disinfectants, phtharal, peracetic acid, glutaral, sodium hypochlorite, and benzalkonium chloride may be effective. Phylogenetic analyses revealed that the strains isolated from salamanders in 2014 and 2018 were genetically closely related, which could indicate an outbreak. The main gross findings in infected salamanders include skin ulcerative lesions or nodules in the enlarged liver. Microscopically, multifocal to coalescent granulomatous lesions composed of massive macrophages containing numerous acid-fast bacilli were prominently observed in the liver.

**Conclusion:**

This study contributes to our understanding of the genetic diversity and phenotypic characteristics of *M. montefiorense*, as well as the pathology of the infection.

## 1. Introduction

Non-tuberculous mycobacteria (NTM) are ubiquitous environmental organisms that can cause infection in humans and animals (1). In amphibians, mycobacteriosis has been associated with NTM species (*Mycobacterium marinum*, *Mycobacterium chelonae*, *Mycobacterium fortuitum*, *Mycobacterium xenopi*, and *Mycobacterium ulcerans* ecovar Liflandii), with infections primarily occurring in frogs in captivity (2–4). *Mycobacterium montefiorense*, a ubiquitous slow-growing NTM, belongs to the *Mycobacterium simiae* complex and is genetically closely related to *Mycobacterium triplex* (5–7). *M. montefiorense* was discovered in captive green moray eels (*Gymnothorax funebris*) and spotted moray eels (*G. moringa*) (8). *M. montefiorense*-associated mycobacteriosis has also been reported in Japanese black salamanders (*Hynobius nigrescens*) and Hakuba salamanders (*H. hidamontanus*) (9).

In Japan, out of the 90 evaluated amphibian species/subspecies, 67 species/subspecies are listed on the “Red List” by the Japanese Ministry of the Environment, and among them, 46 species/subspecies belong to the order *Caudata* (10). Research on the rearing and captive breeding methods and exhibition of these rare amphibians in zoos and aquariums is essential, not only for future conservation efforts but also for raising visitor awareness about the importance of conserving these species (11, 12). Infectious diseases, including *M. montefiorense* infection can damage captive amphibians (9, 13). However, research on *M. montefiorense* and its associated infection is limited.

Phenotypes (i.e., growth rate and temperature, pigment production, colony morphology, and biochemical characteristics) are important factors for mycobacterial species characterization and identification (14, 15). In addition, phenotypic data for antimicrobial susceptibility are crucial for effective antimicrobial treatment strategies (16, 17). Additionally, understanding bacterial susceptibility to disinfectants is critical for quarantine (18). However, *M. montefiorense* phenotypes are less studied. In this study, we evaluated phenotypic characteristics of *M. montefiorense* to provide fundamental phenotypic data.

Though phylogenetic taxonomy using 16S rRNA gene sequencing, which started to dominate the field of bacterial classification in the 1990s, is still a powerful tool, whole-genome sequencing has a higher resolution and can provide insights into exact species and subspecies present (19). In 2018 and 2022, draft genome sequences of *M. montefiorense* strains were reported (9, 20). However, genome-based phylogenetic analysis in detail has not been performed yet. In the present study, we performed phylogentic analyses by using whole-genome sequencing to confirm the phylogenetic relationship and position of *M. montefiorense* among closely related mycobacterial species.

Molecular epidemiological methods using whole genome sequencing data allow tracing transmission chains, identify super-spreaders, and predict undiagnosed transmission events, potentially leading to early treatment of infectious patients and prevention of pathogen spread (21–23). Furthermore, it could assess the effectiveness of intervention strategies for controlling infections (24). In this species, there is no insight into molecular epidemiology. Therefore, we conducted a whole-genome-based molecular epidemiological analysis in *M. montefiorense* strains isolated from 2010 to 2018 to uncover the infectious expansions in the salamander.

Clinical and pathological findings play a key role in the diagnosis (4, 25, 26). Only a few findings (granulomas in the liver and ulcers on the skin) in salamanders infected by *M. montefiorense* were reported (9). We pathologically evaluated infected salamanders with the mycobacterial species to provide more useful evidence/features for diagnosis.

Here, to provide fundamental information regarding the pathogen and pathology of the infection as described above, we evaluated the phenotypic and genetic characteristics of *M. montefiorense*, including strains (*n* = 9) isolated from salamanders, as well as pathology of the salamanders (*n* = 9) infected by *M. montefiorense*. This study will help to develop diagnostic and prevention protocols to control this infection.

## 2. Materials and methods

### 2.1. Bacterial strains

A total of 11 strains, including 9 *M. montefiorense* strains isolated from salamanders, 1 *M. montefiorense* ATCC BAA-256 (reference strain) from a moray eel, and 1 *M. triplex* JCM 14744 (reference strain), were used in this study (Table 1). The salamander strains were collected from the infectious cases in Niigata City Aquarium (Niigata, Japan) between 2010 and 2018, and the isolation and culturing method were as described in the previous papers (9, 20). Briefly, following American Veterinary Medical Association Guidelines for the Euthanasia of Animals (2013 edition) (29), eight dead or euthanized salamanders in the aquarium were collected and routinely dissected. The liver tissues were sampled and frozen at −20°C until further examination. The tissues were thawed, homogenized, and decontaminated with 1 ml of Nacetyl-L-cysteine-sodium citrate-NaOH for no longer than 15 min. After neutralization with 6 mL of phosphate buffer (pH 6.8), the samples were centrifuged at 3,000 × *g* for 20 min; the obtained pellets were then inoculated in Middlebrook 7H10 agar supplemented with 10% BBL Middlebrook oleic acid-albumin-dextrose-catalase (OADC) enrichment (Becton, Dickinson and Company, USA) and in 2% Ogawa egg slants (Kyokuto Pharmaceutical Industrial Co., Ltd., Japan). The media was incubated at 25°C for 2 months. Isolates obtained were identified as *M. montefiorense* based on the Runyon classification system (14), phylogenetic analysis of the 401-bp 65-kDa heat shock protein gene (*hsp*65) amplified with the Tb11/Tb12 primer set (Supplementary Figure S1) (30), and average nucleotide identity analysis using PyANI (31) in their whole-genome sequences.

**Table 1:** Information on the strains used in this study.

Strain	Species	Year isolated	Isolate source	Geographic location	Reference
BS	*Mycobacterium montefiorense*	2010	*Hynobius nigrescens*	Japan	(9)
NJB14191	*M. montefiorense*	2014	*H. hidamontanus*	Japan	(27)
NJB14192	*M. montefiorense*	2014	*H. hidamontanus*	Japan	(27)
NJB14194	*M. montefiorense*	2014	*H. hidamontanus*	Japan	(27)
NJB14195	*M. montefiorense*	2014	*H. hidamontanus*	Japan	(27)
NJB14197	*M. montefiorense*	2014	*H. hidamontanus*	Japan	(27)
NJB18182	*M. montefiorense*	2018	*H. lichenatus*	Japan	(27)
NJB18183	*M. montefiorense*	2018	*H. lichenatus*	Japan	(27)
NJB18185	*M. montefiorense*	2018	*H. lichenatus*	Japan	(27)
ATCC BAA-256	*M. montefiorense*	NC	*Gymnothorax funebris*	USA	(5)
JCM 14744	*M. triplex*	NC	*Homo sapiens*	USA	(28)

NC, not clear.

*Mycobacterium triplex*, a *M. montefiorense* genetically close species, served as a control in several tests.

### 2.2. Microbiology and chemical biology

#### 2.2.1. Preparation of bacterial strains

Strain stocks [stored at −80°C in 20% (v/v) glycerol] were inoculated onto Middlebrook 7H10 agar supplemented with 10% BD BBL™ Middlebrook Oleic Albumin Dextrose Catalase OADC Enrichment (Becton, Dickinson and Company, USA) and pre-cultured for 4 weeks at 25°C. Colonies were suspended in sterile phosphate-buffered saline (PBS) (−), and suspensions were adjusted to an optical density (OD_530_) of 0.08–1.0. These suspensions were used for all experiments unless otherwise noted.

#### 2.2.2. Growth rate and optimal growth temperature

Growth rate and optimal growth temperature were determined following the procedure described by the Japanese Society for Tuberculosis (2016) (32); however, Middlebrook 7H10 agar supplemented with 10% OADC enrichment was used. Suspensions (20 μl) were inoculated on Middlebrook 7H10 agar supplemented with 10% OADC enrichment. The media were incubated at 4, 25, and 37°C for 4 weeks and checked daily for the first week, then once weekly thereafter.

#### 2.2.3. Pigmentation and chemical biology

The pigment production ability of all strains was tested on 2% Ogawa egg slants (Kyokuto Pharmaceutical Industrial Co., Ltd., Japan) as described by Fukano et al. (33). Suspension was inoculated on two 2% Ogawa egg slants and cultured at 25°C for 2 months under dark conditions. One of the slants was irradiated with a 60 W fluorescent lamp at 30 cm for 1 h, and incubated at 25°C for 24 h under dark conditions again. After the procedure, the coloration of the bacterial colonies in the irradiated and non-irradiated slants was compared and pigment production ability was judged according to the Runyon classification system (14).

Urease production and catalase tests were conducted in eight strains (*M. montefiorense* BS, NJB14191, NJB14192, NJB14194, NJB14195, and NJB14197, ATCC BAA-256, and *M. triplex* JCM 14744), according to the procedure described by the Japanese Society for Tuberculosis (2016) (32). In the urease production test, a loopful of 2% Ogawa egg slant-grown colonies was resuspended in 2 mL of 1/100 M phosphate buffer (pH 6.8) supplemented with 3% urea and 0.001% neutral red and incubated at 25°C for 3 days. When the color of the solution changed to red, we interpreted it as positive. For the semiquantitative catalase test, 0.1 mL suspension was inoculated on 2% Ogawa egg slant in a tube of 18 mm × 180 mm and incubated for 3 weeks. After adding 0.5 mL of tween-peroxide solution (prepared by mixing equal volumes of 30% H_2_O_2_ and 10% Tween-80) to the media, the tubes were incubated at approximately 20°C for 5 min. Subsequently, the column of bubbles was measured. The test results were interpreted as follows: ≥45 mm high catalase reaction and < 45 mm low catalase reaction. In thermo-stable catalase test, a loopful of 2% Ogawa egg slant grown colonies was resuspended in 0.5 ml of 1/15 M phosphate buffer (pH 7.0) in test tubes (16 × 125 mm). Afterward, the tubes were incubated at 68°C for 20 min. After cooling, 0.5 ml of the tween-peroxide solution was added and the evolution of bubbles was observed. When the formation of bubbles was observed in 20 min, we interpreted it as a positive reaction.

### 2.3. Antimicrobial and disinfectant susceptibility

Amikacin and ciprofloxacin are used for treating bacterial infections in amphibians (34), and clarithromycin, rifampicin, streptomycin, kanamycin, and doxycycline for NTM diseases in humans (16, 35). Therefore, these seven antimicrobials were subjected to susceptibility tests. Clarithromycin (CAM, FUJIFILM Wako Pure Chemical Corporation, Japan), rifampicin (REF, Sigma-Aldrich, Merck KGaA, Germany), streptomycin (SM, Sigma-Aldrich), kanamycin (KM, Sigma-Aldrich), amikacin (AMK, Sigma-Aldrich), doxycycline (DOXY, Sigma-Aldrich), and ciprofloxacin (CPFX, MP Biomedicals, USA) susceptibilities were determined for the representative four strains (*M. montefiorense* BS, NJB14195, ATCC BAA-256, and *M. triplex* JCM 14744) using the standardized microdilution method for slowly growing mycobacteria as recommended in the Clinical and Laboratory Standards Institute guidelines (36). Minimal inhibitory concentrations (MICs) were determined after incubating inoculated microdilution plates at 25°C for 5 days. The breakpoints of CAM, REF, AMK, DOXY, and CPFX were judged according to the CLSI breakpoint criteria for slowly growing non-tuberculous mycobacteria (36), and those of SM and KM were judged according to the criteria for *M. tuberculosis* (37).

Disinfectant susceptibility was also determined for the four strains, following the methods previously described by Best et al. (38) and Hernández et al. (39). The disinfectants included 2% (W/V) glutaral (STERIHYDE L, Maruishi Pharmaceutical. Co., Ltd., Japan), 0.55% (W/V) phtharal (DISOPA® Solution 0.55%, Johnson & Johnson KK, Japan), 0.3% (W/V) peracetic acid (ACECIDE, Saraya Co. Ltd., Japan), 75% (V/V) ethanol (FUJIFILM Wako Pure Chemical Corporation), sodium hypochlorite (2% of available chlorine concentration) (FUJIFILM Wako Pure Chemical Corporation), 0.5% (W/V) benzalkonium chloride (TEGO 51® Disinfectant Solution 30%, Alfresa Pharma Corporation, Japan), 0.5% (W/V) chlorhexidine gluconate (HIBITANE®, Sumitomo Pharma Co., Ltd., Japan), and 0.13% (W/V) didecyldimethylammonium chloride (Astop, Scientific Feed Laboratory Co., Ltd., Japan). The inoculum suspension (0.1 ml) was adjusted to an optical density (OD_530_) of 0.08–1.0 and was treated with 0.9 ml of the disinfectants and added to 9.9 mL of a neutralizing agent or sterile dilute water. Neutralization or dilution was conducted at 1, 5, 10, 15, 30, and 60 min. Glutaral and phtharal were neutralized by 5% glycine and 0.5% sodium hydrogen sulfite, respectively. Peracetic acid and sodium hypochlorite were neutralized by 3% sodium thiosulfate. All neutralizing agents were purchased from FUJIFILM Wako Pure Chemical Corporation. The neutralized suspension (10 μL) was inoculated on Middlebrook 7H10 agar supplemented with 10% BBL Middlebrook OADC enrichment and incubated at 25°C for 4 weeks. Following incubation, when no colonies were macroscopically observed, we determined the adequate contact time required for disinfection. Disinfectant susceptibility was tested in duplicate, and the longer time was adopted as the adequate contact time.

### 2.4. Phylogenetic analyses

#### 2.4.1. Core genome MLST analysis

Core genome multilocus sequence typing (cgMLST) analysis was performed in 29 *Mycobacterium* spp. strain set from the National Center for Biotechnology Information (NCBI) database, including nine salamander strains (9, 20) and the reference strain (*M. montefiorense* DSM 44602). The analysis was performed following the pipeline described by Atxaerandio-Landa et al. (40). Specifically, the assembled genome sequences from the NCBI database were assessed using CheckM taxonomy_wf v1.2.0 + galaxy0 (--rank genus *Mycobacterium*) (28). The sequences, assessed as >99% completeness and < 5% contamination, were reannotated using Prokka v1.14.6 (41), and general feature format (gff) files were produced. The gff files were analyzed using Roary v3.13.0 (42) for core genes. A maximum likelihood tree was constructed from the core gene alignment using the best-fitted nucleotide substitution model (the 29 *Mycobacterium* spp. strain set, GTR+ F + I + G4; the *M. montefiorense* strain set, GTR + F) in the IQtree web server^1^ (43) and visualized with Interactive Tree of Life (iTOL).^2^

#### 2.4.2. Linkage network analysis

**Figure 1:**
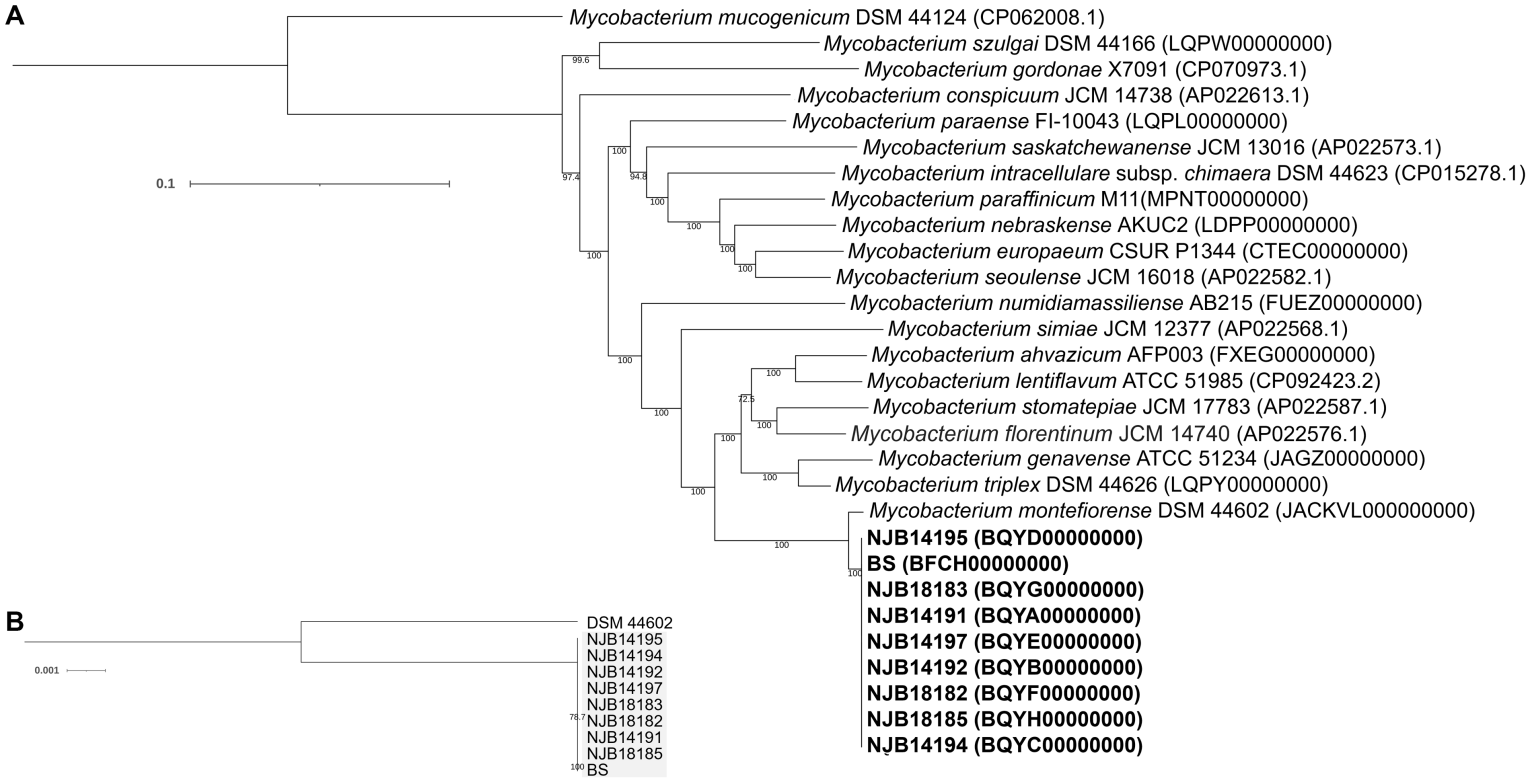
Phylogenetic analysis of *Mycobacterium montefiorense*. **(A,B)** Maximum likelihood phylogenies in the 29 *Mycobacterium* spp. strains and the *M. montefiorense* strains. The tree is midpoint rooted. Scale bars indicate nucleotide diversity.

Linkage network analysis (23, 44) using core single nucleotide polymorphisms (SNPs) in the ten *M. montefiorense* strains was performed, based on clustering in the maximum likelihood phylogeny. *M. montefiorense* ATCC BAA-256 was used as an outgroup strain. Briefly, short-read sequencing data from nine strains of *M. montefiorense*, including strains from salamanders, were obtained from the NCBI database (Supplementary Table S1). The quality of raw reads was assessed with FastQC v0.11.9 (45). Core SNPs were called from the fastp-trimmed data with Snippy v4.6.0 + galaxy0 using the draft genome of *M. montefiorense* BS (5,744,567 bp, BFCH00000000) as a reference, and core SNP alignment, including the reference, was then generated using Snippy-core v4.6.0 + galaxy0.^3^ The reference was selected based on the quality of sequences (Supplementary Table S1). From the core SNP alignment, pairwise SNP distances were calculated. Subsequently, a median joining network (46) was generated based on the SNPs of the core alignment in pop art v1.7 (47). Furthermore, to improve the resolution of the analysis, a median-joining analysis in the salamander strains within the same cluster (Figure 1A) was also conducted. Default parameters were used for all software unless otherwise noted.

### 2.5. Pathology

Salamanders, including Hakuba salamanders (*n* = 5) and Tohoku hynobiid salamanders (*n* = 4), that had been reared and exhibited in the Niigata City Aquarium and diagnosed as *M. montefiorense* infection, were subjected to histopathological examination. Of them, the salamander strains NJB14191, NJB14192, NJB14194, NJB14195, NJB14197, NJB18182, NJB18183, and NJB18185 were isolated (see Supplementary Table S2) (20). The examined salamanders in this study were hatched from wild-collected eggs and were reared in the aquarium (Supplementary Appendix S1; Supplementary Figure S2).

The salamanders were routinely dissected, and their external and internal gross features (reproductive organ, and/or alimentary tract) and fixed in a 10% phosphate-buffered formalin solution. The formalin-fixed tissues were processed routinely to prepare paraffin sections for histopathology. The sections were stained with hematoxylin and eosin (H&E) and Ziehl–Neelsen (ZN) stains.

## 3. Results

### 3.1. Microbiology and chemical biology

In growth rate and temperature, pigmentation, and chemical biology tests, the *M. montefiorense* strains from salamanders showed characteristics similar to those of *M. montefiorense* ATCC BAA-256 (Table 2).

**Table 2:** Microbiological and chemical characteristics.

	Date isolated	Source	Growth rate	Growth temperature			Colony	Pigmentation	Catalase test	Catalase test (68°C)	Urease production test
				4°C	25°C	37°C					
Mm BS	2010	Salamander	SG	−	+	+	S	NP	L	−	−
Mm NJB14191	2014	Salamander	SG	+	+	+	S	NP	L	−	−
Mm NJB14192	2014	Salamander	SG	+	+	+	S	NP	L	−	−
Mm NJB14194	2014	Salamander	SG	+	+	+	S	NP	L	−	−
Mm NJB14195	2014	Salamander	SG	+	+	+	S	NP	L	−	−
Mm NJB14197	2014	Salamander	SG	+	+	−	S	NP	L	−	−
Mm NJB18182	2018	Salamander	SG	+	+	+	S	NP	ND	ND	ND
Mm NJB18183	2018	Salamander	SG	+	+	+	S	NP	ND	ND	ND
Mm NJB18185	2018	Salamander	SG	+	+	+	S	NP	ND	ND	ND
Mm ATCC BAA-256	NC	Fish	SG	+	+	+	S	NP	L	−	−
Mt JCM 14744	NC	Human	SG	+	+	+	S	NP	L	+	+

Mm, *Mycobacterium montefiorense*; Mt, M. triplex; SG, slow growing; S, smooth; L, low catalase reaction; NP, non-photochromogenic; ND, not done; NC, not clear.

### 3.2. Antimicrobial and disinfectant susceptibility

MICs of seven antimicrobials are summarized in Table 3, and all strains of *M. montefiorense* were susceptible to CAM according to the CLSI breakpoint criteria (36). Against the other six antimicrobials (REF, SM, KM, AMK, DOX, CPFX), *M. montefiorense* strains showed intermediate resistance, according to the criteria (36, 37).

**Table 3:** Minimum inhibitory concentrations (MICs) of antimicrobials against mycobacterial species.

Strain	Antimicrobial*						
	CAM	REF	SM	KM	AMK	DOXY	CPFX
Mm BS	4 (S)	>32 (R)	128 (R)	8 (R)	>64 (R)	>128 (R)	4 (R)
Mm NJB14195	2 (S)	32 (R)	64 (R)	32 (R)	64 (R)	>128 (R)	4 (R)
Mm ATCC BAA-256	2 (S)	>32 (R)	8 (R)	8 (R)	32 (I)	>128 (R)	4 (R)
Mt JCM 14744	16 (I)	>32 (R)	32 (R)	>128 (R)	>64 (R)	>128 (R)	>32 (R)

*Minimum inhibitory concentrations (μg/ml). Mm, *Mycobacterium montefiorense*; Mt, M. triplex; CAM, clarithromycin; REF, rifampicin; SM, streptomycin; KM, kanamycin; AMK, amikacin; DOXY, doxycycline; CPFX, ciprofloxacin S, susceptible; I, intermediate; R, resistant: The breakpoints of CAM, REF, AMK, DOXY, and CPFX were judged according to the CLSI breakpoint criteria of slowly growing non-tuberculous mycobacteria (45), and those of SM and KM were according to the criteria in *M. tuberculosis* (46).

Adequate contact times of eight disinfectants are listed in Table 4. Four *M. montefiorense* strains were disinfected with phtharal and peracetic acid for ≤5 min, and with glutaral, sodium hypochlorite, and benzalkonium chloride for ≤60 min. Ethanol, didecyldimethylammonium chloride, and chlorhexidine gluconate did not disinfect certain strains with ≤60 min exposure.

**Table 4:** Adequate contact time of disinfectants for mycobacterial species.

	Glutaral	Phtharal	Peracetic acid	Ethanol	Sodium hypochlorite	Benzalkonium chloride	Chlorhexidine gluconate	Didecyldimethylammonium chloride
Mm BS	15 < t ≤ 30	<1	1 < t ≤ 5	>60	30 < t ≤ 60	30 < t ≤ 60	>60	>60
Mm NJB14195	10 < t ≤ 15	<1	<1	>60	1 < t ≤ 5	10 < t ≤ 15	>60	>60
Mm ATCC BAA-256	30 < t ≤ 60	<1	<1	>60	15 < t ≤ 30	15 < t ≤ 30	>60	>60
Mt JCM 14744	>60	1 < t ≤ 5	1 < t ≤ 5	>60	15 < t ≤ 30	>60	>60	>60

Mm, *Mycobacterium montefiorense*; Mt, M. triplex; t, minimum time (min) required to disinfect (no colony grew after treatment).

### 3.3. Phylogenetic analyses

**Figure 2:**
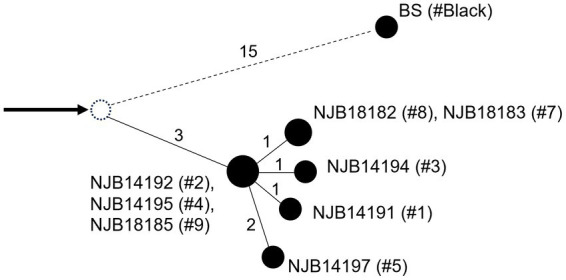
Median joining network in the 10 *Mycobacterium montefiorense* strains. Single nucleotide polymorphisms (SNPs) between strains are shown by numbers. Arrow denotes an outgroup strain. A dashed circle denotes a hypothetical common ancestor. # Denotes the ID numbers of the salamanders.

The cgMLST phylogeny based on 92 core genes (83,316 bp) showed that *M. montefiorense* was most closely related to *M. triplex* (Figure 1A). The cgMLST analysis based on the 4,630 core genes (4,669,478 bp) in the *M. montefiorense* strains indicated that the strains from isolated salamanders were classified into the same cluster, separated from the fish strain (Figure 1B). For pairwise SNP distances in the *M. montefiorense* strains, a total of 88,507 SNP loci were detected among 5,114,752 bp of the alignment of the *M. montefiorense* strain sequences. In the salamander strains, ≤15 SNPs were present, and the intragroup variation of the strains isolated in 2014 and in 2018 was 0–3 SNPs (see Supplementary Table S3). In the median joining network, BS and salamander strains (NJB14192, NJB14195, and NJB18185) show an accumulation of unique 15 SNPs and 3 SNPs, respectively, from a hypothetical common ancestor (Figure 2). Furthermore, from the three salamander strains, the other strains show an accumulation of unique 1–2 SNPs.

In addition, for pairwise SNP distances in a broader range of core regions using the salamander strain set, a total of 23 SNP loci were detected in 5,463,489 bp of the alignment of the salamander strain sequences (Supplementary Table S4; Supplementary Figure S1). Strain BS varied from the other salamander strains by 18–20 SNPs, and the other strains had 0–3 SNPs between each other.

### 3.4. Clinical signs and gross features

The dead salamanders showed only loss of energy and anorexia on the day before death. Six diseased Hakuba and Tohoku hynobiid salamanders showed ulcerative skin lesions on the neck, extremities, body side, and/or tail (Table 5). The livers of six salamanders were enlarged. In the three Hakuba salamanders, the livers were associated with multiple or coalescent gray-to-black nodules. In the livers of Tohoku hynobiid salamanders, multiple coalescent tan to white nodules were observed. Splenomegaly with multifocal-coalescent white nodules was also seen in five salamanders.

**Table 5:** Histopathology data.

No.	species	Date collected	Dead/ euthanized	Gross lesions	Histopathology					
					L	Sp	AT	Sk	K	RO
1	*Hynobius hidamontanus*	10/20/2014	Euthanized	Skin ulcer in the dorsal side of the tail and left lateral neck. Hepatomegaly with multifocal-coalescent gray-black nodules. Splenomegaly with multifocal-coalescent white nodules.	+	NE	−	+	NE	−
2	*H. hidamontanus*	10/20/2014	Euthanized	Skin ulcer in the arms and right leg. Hepatomegaly with multifocal-coalescent gray-black nodules. Splenomegaly with multifocal-coalescent white nodules	+	NE	−	+	NE	−
3	*H. hidamontanus*	10/20/2014	Euthanized	Skin ulcer in the right forearm. Hepatomegaly with multifocal-coalescent gray-black nodules. Multifocal white nodules in the spleen	+	NE	−	−	NE	−
4	*H. hidamontanus*	10/20/2014	Euthanized	Skin ulcer in the ventral side of the abdomen. Hepatomegaly with multifocal-coalescent gray-black nodules.	+	NE	−	+	NE	−
5	*H. hidamontanus*	10/20/2014	Euthanized	−	+	NE	−	NE	NE	−
6	*Hynobius lichenatus*	3/28/2018	Dead	Hepatomegaly with multifocal-coalescent white nodules. Splenomegaly with multifocal-coalescent white nodules	+	+	+	+	+	+
7	*H. lichenatus*	8/26/2018	Dead	Skin ulcer in the dorsal side of the tail. Multifocal-coalescent gray-black nodules in the liver. Splenomegaly with multifocal white nodules.	+	+	+	+	+	+
8	*H. lichenatus*	8/28/2018	Euthanized	−	−	−	−	−	−	−
9	*H. lichenatus*	8/28/2018	Dead	Skin ulcer in the right foot. Hepatomegaly with multifocal-coalescent white nodules. Splenomegaly with multifocal- coalescent white nodules.	+	+	+	+	+	+

NE, not examined; NA, not applicable; L, liver; Sp, spleen; AT, alimentary tract; Sk, skin; K, kidney; RO, Reproductive organ. + Acid-fast bacilli were observed.

### 3.5. Histopathology

**Figure 3:**
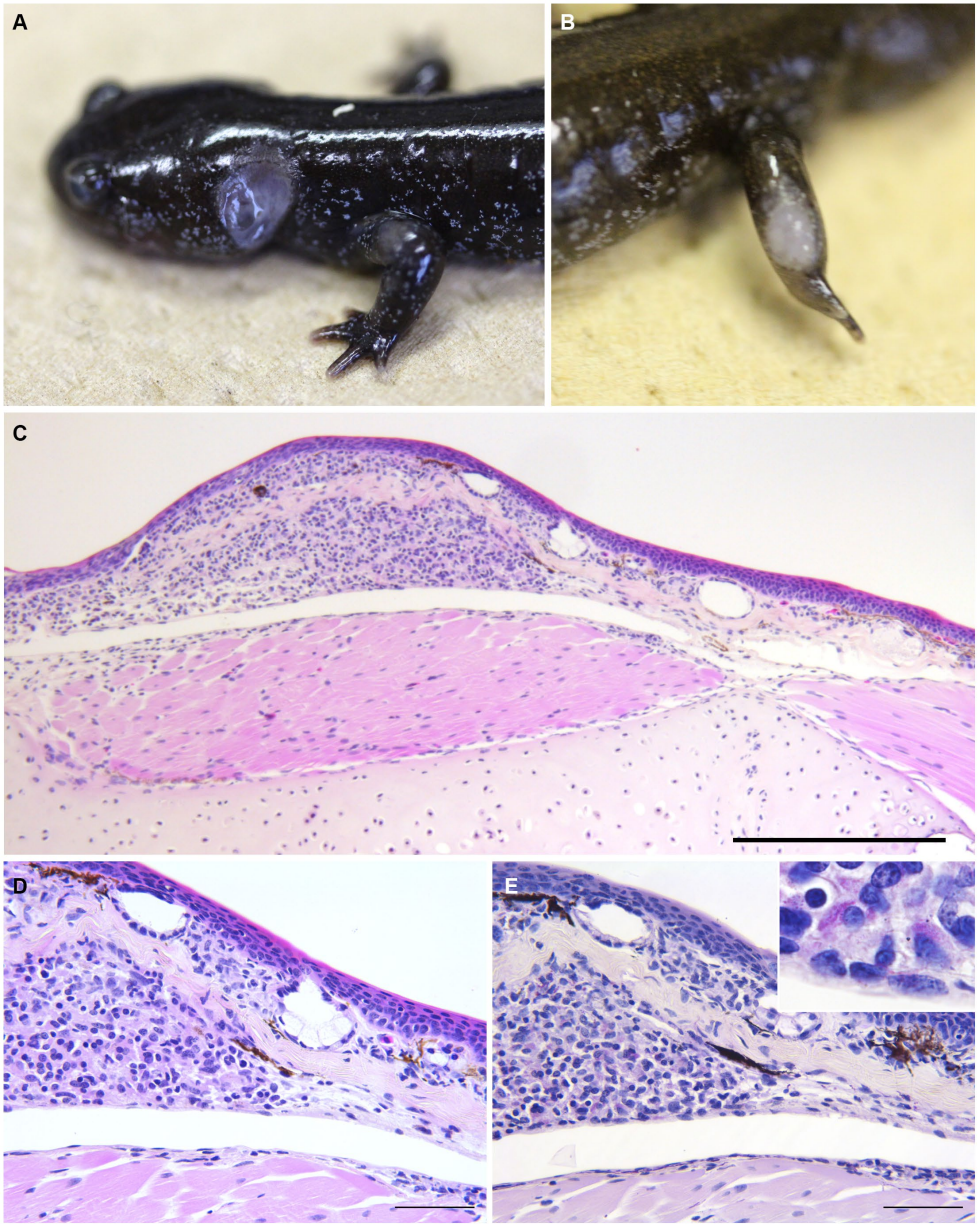
Gross and histopathological skin lesion in infected salamanders. **(A,B)** Skin legion in Hakuba salamanders (**A**, #1; **B**, #3). **(C,D)** The skin lesion of Hakuba salamander #4. Granulomas composed of aggregates of round to polygonal macrophages with abundant pale staining or occasional foamy cytoplasm and an eccentric round to ovoid nucleus, mixed with few lymphocytes and granulocytes were observed from the dermis to subcutaneous tissue (**C**, H&E stain, scale bar = 500 μm; **D**, H&E stain, scale bar = 100 μm). **(E)** Macrophages contain numerous acid-fast bacilli (ZN stain, scale bar = 100 μm).

The most prominent histopathological features included multifocal to coalescent granulomatous lesions observed in the livers of eight salamanders with numerous acid-fast bacilli (Figure 2). Granulomas exist in the hepatic parenchyma and hematopoietic tissue of the liver, and most of the hepatic parenchyma was affected by the multifocal to coalescent lesions replacing normal parenchyma. These granulomas were characterized by aggregates of round to polygonal macrophages with abundant pale staining or occasional foamy cytoplasm and an eccentric round-to-ovoid nucleus, mixed with few lymphocytes and granulocytes. Numerous 1.5–3 × 0.3–0.5-μm sized acid-fast bacilli were often present within the cytoplasm of macrophages. In three salamanders, mild to severe multifocal granulomas with numerous acid-fast bacilli were observed in the systemic organs (i.e., skin, spleen, liver, kidney, reproductive organ, and alimentary tract) (Table 5). Granulomas in the splenic parenchyma were also seen. In the skin lesion, the granulomas were observed from the dermis to subcutaneous tissue (Figure 3). In a salamander that did not have the ulcer lesions, some macrophages with acid-fast bacilli were sporadically present in the dermis. In the kidney, the granulomas were located in the interstitium, and extracellular acid-fast bacterial colonies and bacteria within tubular epithelial cells were observed. In addition, macrophages containing acid-fast bacilli were also detected in the lamina propria to serosa in the alimentary tract and the lumen, lamina propria, and serosa of the oviduct. In a Tohoku hynobiid salamander, occasional multinucleated giant cells (Langhans type) were also observed in the liver. In one case, a granuloma was associated with a necrotic center containing numerous acid-fast bacilli.

## 4. Discussion

**Figure 4:**
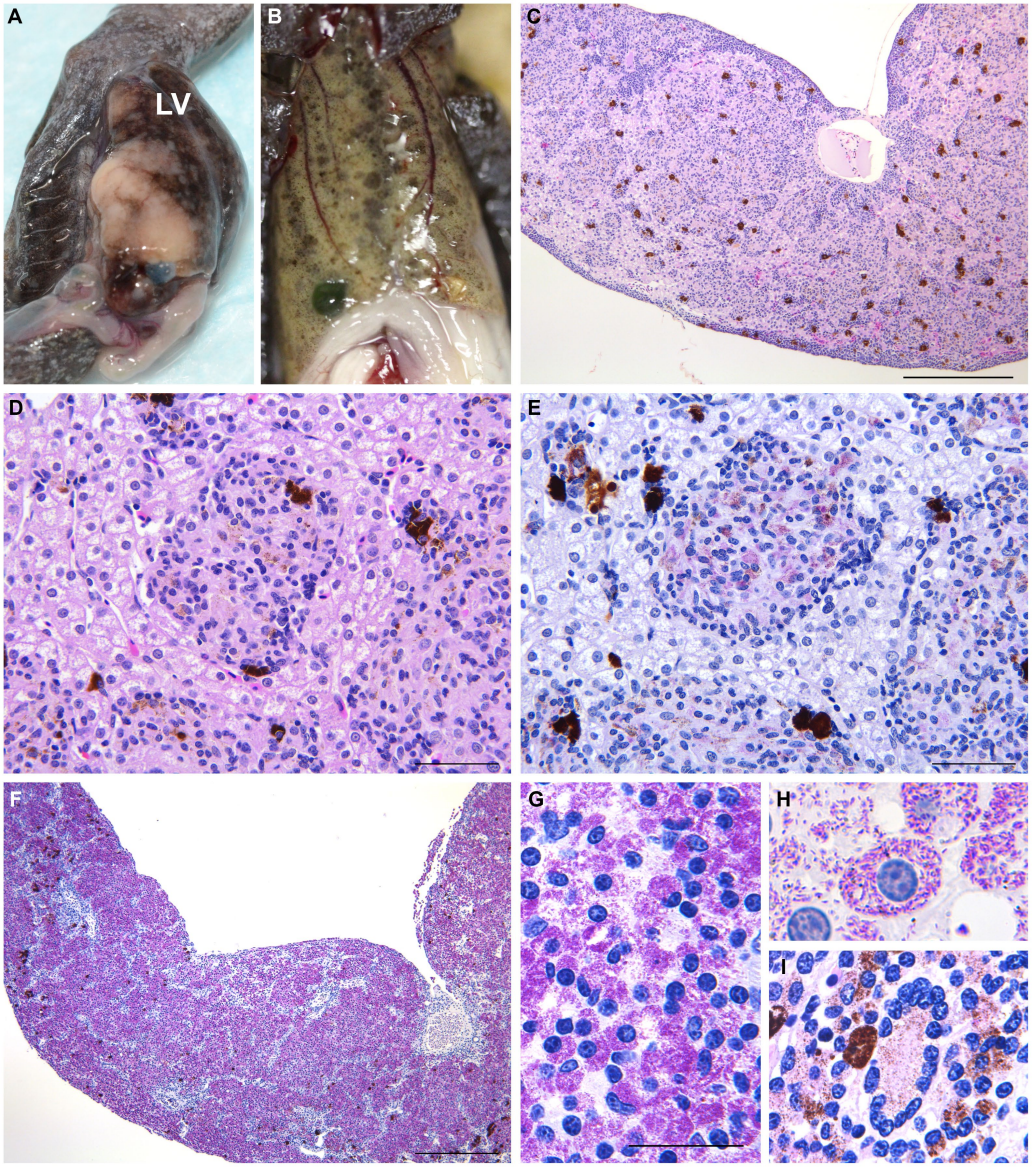
Gross and histopathological legion in the liver in infected salamanders. **(A)** Variably sized granulomatous nodules within the liver of a Tohoku hynobiid salamander #9. LV, liver. **(B)** Gray to black nodules in the liver of a Tohoku hynobiid salamander #3. **(C,D)** Multifocal nodular aggregates of histiocytes, resulting in hepatocyte compression and atrophy, were observed in a Hakuba salamander (#4). These granulomas were composed of aggregates of round to polygonal macrophages with abundant pale staining or occasional foamy cytoplasm and an eccentric round to ovoid nucleus, mixed with few lymphocytes and granulocytes (**C**, H&E stain, scale bar = 500 μm; **D**, H&E stain, scale bar = 100 μm). **(E)** Numerous acid-fast bacilli observed within the macrophages (ZN stain, scale bar = 100 μm). **(F)** More than 90% of the parenchyma is affected by coalescent granulomatous lesions replacing normal hepatic parenchyma in salamander #9. **(G,H)** Acid-fast bacilli 1.5–3 × 0.3–0.5 μm in size (**G**, ZN stain, scale bar = 50 μm; **H**, ZN stain, ×1,000). **(I)** Multinucleated giant cell observed in the liver of Tohoku hynobiid salamander #6 (ZN stain, ×400).

*Mycobacterium montefiorense*-caused mycobacteriosis in moray eels was first reported in 2001, where the pathogen was characterized based on molecular biological analyses for several genes, mycolic acids, and phenotypic data such as growth and biochemical composition (5, 8). Although Fukano et al. and Komine et al. reported the draft genome of the *M. montefiorense* strains from salamander species (9, 13), the pathology- and phenotype-related information was limited for the case. This is the first report on the pathology of mycobacteriosis associated with *M. montefiorense* in salamanders, along with detailed phenotypic characteristics such as drug susceptibility and genetic characteristics of this pathogen. Microbiological and chemical characteristic evaluations were similar between the isolated strains from salamanders and the type strain isolated from a moray eel, although the salamander strains varied from the eel strain by at least 88,000 SNPs. Therefore, these phenotypic characteristics could be stable in *M. montefiorense*, and are useful to identify this mycobacterium species (Figure 4).

As there are no effective treatments for mycobacterial infections in amphibians and there is a risk of zoonosis, euthanasia is recommended (3). However, *in vitro* susceptibility testing in this study has suggested that CAM could be effective against mycobacteriosis associated with *M. montefiorense* and other species of the *M. simiae* complex, despite the high levels of observed natural resistance (48). Similar to other veterinary diseases with zoonotic potential, attempting treatment with caution, as long as personnel administering treatment apply correct personal protection, is plausible. However, to our knowledge, no cases of CAM use have been reported in amphibians. Further study into the clinical effectiveness and side effects of CAM treatment would therefore be required.

NTM are ubiquitous agents that are isolated from environmental sources, and *M. montefiorense* has been isolated from water, aquatic plants, and sediment from ponds (6). Reducing pathogen numbers in patient environments using disinfection could prevent infectious diseases (1, 49). However, *Mycobacterium* spp. is known to pose resistance to disinfectants, and an intermediate- or high-level disinfectant is generally required (18, 50, 51). The disinfectant susceptibility testing in this study suggested the effectiveness of three high-level disinfectants: phtharal and peracetic acid in 5 min and glutaral in 60 min, as well as sodium hypochlorite, an intermediate-level disinfectant in 60 min and benzalkonium chloride, a low-level disinfectant, in 60 min. These time durations are similar to those recommended for disinfection of *M. tuberculosis* (18). It should be noted that *M. montefiorense* is highly resistant to ethanol, which is often used to disinfect equipment; therefore, ethanol is not considered appropriate for the disinfection of *M. montefiorense*.

In this study, the cgMLST phylogeny based on the 92 core genes confirmed that *M. montefiorense* is genetically closely related to *M. triplex*, supporting the insights of the previous papers (5–7, 20). Furthermore, the phylogeny of the 4,630 core genes suggested that the salamander strains form a cluster that is separated from a fish strain (ATCC BAA-256).

SNP analysis using whole-genome sequences has currently the highest level of resolution and is used for tracking transmission (23, 44, 52, 53). In the linkage network analysis of the *M. montefiorense* strains including ATCC BAA-256 as an outgroup, intragroup variation of strains within the cluster in the cgMLST analysis was ≤15 SNPs. The intragroup variation of the strains isolated in 2014 and in 2018 was 0–3 SNPs. There are no studies on the definition of a genetic relatedness cut-off (SNP values) and mutation rate in DNA sequences per genome per year in *M. montefiorense*. However, a cut-off value of 12 SNPs is often used and it has been suggested that the rate of genetic changes is approximately 0.5 SNPs per genome per year in latent, active, as well as re-activated diseases (21, 54, 55). A study of *M. chimaera* revealed a maximum of 38 SNPs among strains related to an outbreak (56). Specifically, 20–30 SNPs are defined as a cut-off value for transmission in *M. abscessus*, a rapidly growing mycobacterium (57–59). In addition, given that ≤3 SNPs were present in the strains isolated from salamanders collected on the same day in 2014 that had been reared in the same place and 0–3 SNPs were found between the strains in 2014 and the strains in 2018; therefore, these strains could be regarded as “possibly related.” This molecular epidemiological result supported the epidemiological suggestion that there is a possible epidemiological link between the salamanders in 2014 and 2018 (see Supplementary Appendix S1; Supplementary Figure S2). We therefore estimated the situation in 2014 and 2018 could be an outbreak. Moreover, we speculated that the infection could have spread from individuals #2, #4, and #9 to individuals #1, #3, #5, #7, and #8 in chronological order, although the transmission route is unclear. In the aquarium, infected salamanders were identified and removed, and disinfection of the equipment suspected to be contaminated by the pathogen with boiling water was conducted as an intervention to control this infection from 2012. Since 2019, no infection in salamanders has occurred. This indicates that basic infection control strategies, such as the detection and removal of infected animals and disinfection of rearing facilities and the environment, were potentially effective against this infection. However, because of the difficulty in detecting infected individuals, where specific clinical signs are lacking, it could take a long period (several years) for the infection situation to resolve. Molecular epidemiological approaches with whole genome sequencing are now contributing to the control of infectious diseases in humans, enabling the identification of transmission routes and sources and the evaluation of interventions (21–24, 60–62). This study suggests that the use of molecular epidemiological approaches could contribute to controlling infections, in zoos and aquariums as well.

In amphibian mycobacteriosis, nodules are generally formed on the skin and subcutaneous lymph sacs, with abscess formation and ulceration. In abdominal organs, white to yellow nodules are locally or multifocally observed, especially in the liver, spleen, and kidney (4, 63–66). Histologically, multifocal to coalescent granulomas were observed in the salamander organs affected by mycobacteria. Granulomas are commonly composed of large macrophages (epithelial cells), mixed with few neutrophils, eosinophils, and lymphocytes. In some cases, a few multinucleated giant cells were present. Serous granuloma formation can be seen in chronic lesions or cases caused by mycolactone-producing mycobacteria (i.e., *M. ulcerans* ecovar Liflandii). Chronic granulomas are surrounded by fibrous capsules, and acid-fast bacilli are often found within macrophages (4, 63–66). Although the granulomas of the salamanders in this study had no fibrous capsules, the gross and pathological features were generally consistent with the findings commonly seen in mycobacteriosis of amphibians, as described above. Furthermore, our histopathological findings were similar to those of *M. montefiorense* infections in eels (8). These findings suggest that it is difficult to determine *M. montefiorense* as the causative agent of mycobacteriosis from pathological findings alone. Additionally, the detection of pathogenic genes from lesions and/or cultures of pathogens is necessary for diagnosis.

This study has a few limitations. First, this was a single-site study with a limited sample size. Second, the *in vivo* efficiency of antimicrobials in salamanders is uncertain, as only *in vitro* susceptibility testing for antimicrobials was conducted and the interpretation of the results (susceptible, intermediate, resistant) was based on the CLSI breakpoint criteria for humans. Third, the cut-off value for transmission and genome mutation rate per year in *M. montefiorense* remains uncertain. In this study, based on that among the strains isolated from tank-shared salamanders in 2014 and that of the other slowly growing mycobacteria, we judged the number of SNPs to be relevant at ≤3 (21, 54–56). Fourth, intrusion and transmission routes (salamander to salamander/environmental sources to salamander) were unclear. To address these limitations, the accumulation of infectious cases and further studies on the *in vivo* effectiveness of antimicrobials and molecular epidemiology in *M. montefiorense* are required. Nevertheless, we believe that this study provides novel and essential data on *M. montefiorense* and the associated infection.

In conclusion, this study characterized the mycobacterial strains isolated from salamanders based on phenotypic and genetic examination and the pathology of mycobacteriosis infection by *M. montefiorense* in salamanders. The study provides valuable information to diagnose and handle *M. montefiorense* infection as follows:

1. Microbiological and chemical characteristic evaluation findings were similar between the isolated strains (from salamanders) and the type strain (isolated from a moray eel).
2. Susceptibility testing for antimicrobials suggested that CAM-based treatment may be effective.
3. Disinfectant susceptibility testing suggested that phtharal, peracetic acid, glutaral, sodium hypochlorite, and benzalkonium chloride may be effective.
4. Phylogenetic analyses revealed that *M. montefiorense* strains isolated from the salamanders between 2014 and 2018 were genetically closely related, which could indicate an outbreak.
5. The main findings include skin ulcerative lesions or nodules in the enlarged liver in infected salamanders.
6. Multifocal to coalescent granulomatous lesions composed of massive macrophages containing numerous acid-fast bacilli in the liver were prominently observed.

This study will help clarify the genetic diversity and phenotypic characteristics of *M. montefiorense*, as well as the pathology of the associated infection.

## Data availability statement

The datasets presented in this study can be found in online repositories. The names of the repository/repositories and accession number(s) can be found in the article/Supplementary material.

## Ethics statement

The animal study was approved by National Institute of Infectious Diseases (NIID) Institutional Animal Care and Use Committee. The study was conducted in accordance with the local legislation and institutional requirements.

## Author contributions

HF and SW conceptualized the manuscript. TK wrote the original draft preparation. TK, HIh, MI, JK, AS, HF, AM, TT, and SS performed the pathological examination, microbiological and molecular analyses. HIw and SH managed the animals and collected the samples and epidemiological data. MY and YH revised the study. All authors read and approved the submitted version.

## Footnotes

^1^https://iqtree.cibiv.univie.ac.at

^2^https://itol.embl.de/

^3^https://github.com/tseemann/snippy
