# Supplementary material for: Non-tuberculous mycobacterial disease associated with Mycobacterium montefiorense in salamanders
Source: Front Vet Sci. 2023 Oct 26;10:1248288. doi: 10.3389/fvets.2023.1248288 (PMC10637390; doi:10.3389/fvets.2023.1248288)
Supplement: Supplementary file 1 [file Data_Sheet_1.docx]

Supplementary Material

# Supplementary Data Index

S1 Appendix: History of salamanders

S1 Materials & Methods: *Hsp65* sequencing and phylogenetic analyses

S1 Figure: (a) Phylogenetic tree of the *hsp65* gene in *M. montefiorense* and the other *Mycobacterium* spp. The tree was inferred with the neighbor-joining method using the Kimura 2-parameter method. Bootstrap values are indicated at nodes as a percentage of 1,000 replicates. Scale bar indicates nucleotide divergence (b) Median joining network based on core SNPs in a nine *M. montefiorense* salamander strain set.

S2 Figure: (a) Epidemiological data of respective individuals. (b) The number of captive Hakuba salamanders and cause of death in them in Niigata City Aquarium between 2008 and 2022. (c) The number of captive Tohoku hynobiid salamanders and cause of death in them in Niigata City Aquarium between 2008 and 2022.

S1 Table: Sequencing data of the *Mycobacterium montefiorense* strains.

S2 Table: Results of pathological and genetic examinations and culture in salamanders examined.

S3 Table: Single nucleotide polymorphism (SNP) matrix in 10 strains of *Mycobacterium montefiorense*.

S4 Table. Single nucleotide polymorphisms (SNPs) matrix in 9 strains of *Mycobacterium montefiorense*

# Supplementary Appendix

**S1 Appendix. History of salamanders**

・Salamander larvae were hatched from wild-collected eggs that were raised in the aquarium.

・Three salamander species (*H. nigrescens*, *H. hidamontanus*, and *H. lichenatus*) were reared in separated tanks that were closely located in the facility.

・Though there was no conscious contact of animals between the different tanks, a possibility that equipment was shared between the tanks remains.

・Overlapping periods existed with rearing the salamanders.

・Salamanders with liver nodules started to be observed from 2008.

・Salamanders infected with *M. montefiorense* were identified and removed (treatments were not performed).

・Tanks where the infection occurred were disinfected with boiling water and the tanks soil was changed.

・Since 2019, no infection associated with *M. montefiorense* has occurred.

# Supplementary Materials & Methods

***Hsp65* sequencing and phylogenetic analyses**

Phylogenetic analysis of the *hsp65* gene, core genome multilocus sequence typing, and core single nucleotide polymorphism (SNP) analysis were conducted to determine relationships among *M. montefiorense* strains. Genomic DNA was extracted from the *M. montefiorense* strains as per the boil method as described by Komine et al. (1). The 401-bp *hsp65* gene was determined using the primer set Tb11–Tb12 (2) and the cycle described by Antuofermo et al. (3). The PCR mixture (20 μL) contained 11.3 μL H_2_O, 4.0 µL 5X colorless buffer, 1.6 µL 2.5 mM dNTP, 1.0 µL 10 μM forward primer, 1.0 µL 10 μM reverse primer, 0.1 µL GoTaq^®^ DNA polymerase (Promega Corp., Madison, WI, USA), and 1 µL template DNA. The amplicons were purified using NucleoSpin^®^ Gel and PCR Clean-up (MachereyNagel GmbH & Co. KG, Germany). The purified amplicons were sequenced with capillary sequencers (Applied Biosystems 3130xl Genetic Analyzer or Applied Biosystems 3730xl DNA Analyzer, Applied Biosystems Inc., Foster City, CA, USA). Sequences were aligned using ClustalW in MEGA X (4), and the sequence similarities were analyzed through BLAST (GenBank, <http://www.ncbi.nlm.nih.gov/blast.cgi>). Subsequently, the neighbor-joining tree of the 401-bp *hsp65* gene from *M. montefiorense* strains as well as mycobacterial strains from the NCBI database was constructed based on Kimura’s two-parameter model and 1,000 bootstrap replicates in MEGA X.

# Supplementary Table

**S1 Table. Sequencing data of the *Mycobacterium* spp. strains**

| Strain | No. of  raw reads | DRA accession no. | Assembly |  |  |  |  |  |  |
| --- | --- | --- | --- | --- | --- | --- | --- | --- | --- |
|  |  |  | Total length | No. of sequences | N50 | Complet-eness | Contamination |  | Genome accession no. |
| *Mycobacterium montefiorense* BS | NA | NA | 5744567 | 50 | 416792 | 99.61 | 1.38 |  | BFCH00000000 |
| *Mycobacterium montefiorense* NJB14191 | 3,646,896 | DRR357474 | 5744673 | 123 | 171591 | 99.53 | 1.38 |  | BQYA00000000 |
| *Mycobacterium montefiorense* NJB14192 | 1,841,220 | DRR357475 | 5776754 | 234 | 157068 | 99.44 | 1.69 |  | BQYB00000000 |
| *Mycobacterium montefiorense* NJB14194 | 1,946,204 | DRR357476 | 5753077 | 155 | 169431 | 99.25 | 1.52 |  | BQYC00000000 |
| *Mycobacterium montefiorense* NJB14195 | 2,424,346 | DRR357477 | 5749641 | 116 | 201183 | 99.19 | 1.67 |  | BQYD00000000 |
| *Mycobacterium montefiorense* NJB14197 | 3,047,344 | DRR357478 | 5745062 | 108 | 201635 | 99.57 | 1.38 |  | BQYE00000000 |
| *Mycobacterium montefiorense* NJB18182 | 2,308,180 | DRR357479 | 5764439 | 210 | 136848 | 99.58 | 2.16 |  | BQYF00000000 |
| *Mycobacterium montefiorense* NJB18183 | 2,811,918 | DRR357480 | 5749386 | 127 | 207711 | 99.49 | 1.38 |  | BQYG00000000 |
| *Mycobacterium montefiorense* NJB18185 | 4,174,608 | DRR357481 | 5739217 | 88 | 240008 | 99.61 | 1.38 |  | BQYH00000000 |
| *Mycobacterium montefiorense* ATCC BAA-256 | 8,308,120 | DRR361296 | 5226877 | 735 | 16108 | 81.71 | 2.06 |  | BSAJ00000000 |
| *Mycobacterium montefiorense* DSM 44602 | NA | NA | 5632745 | 18 | 737684 | 99.53 | 1.11 |  | JACKVL000000000 |
| *Mycobacterium simiae* JCM 12377 | NA | NA | 5788994 | 1 | 5788994 | 99.61 | 1.78 |  | AP022568.1 |
| *Mycobacterium saskatchewanense* JCM 13016 | NA | NA | 6008916 | 1 | 6008916 | 100 | 1.81 |  | AP022573.1 |
| *Mycobacterium florentinum* JCM 14740 | NA | NA | 6219859 | 1 | 6219859 | 99.61 | 1.5 |  | AP022576.1 |
| *Mycobacterium seoulense* JCM 16018 | NA | NA | 5531300 | 1 | 5531300 | 99.94 | 1.11 |  | AP022582.1 |
| *Mycobacterium stomatepiae* JCM 17783 | NA | NA | 6210822 | 1 | 6210822 | 99.61 | 2.08 |  | AP022587.1 |
| *Mycobacterium conspicuum* JCM 14738 | NA | NA | 6237139 | 1 | 6237139 | 99.11 | 0.49 |  | AP022613.1 |
| *Mycobacterium intracellulare* subsp. *chimaera* DSM 44623 | NA | NA | 5865644 | 1 | 5865644 | 99.94 | 1.04 |  | CP015278.1 |
| *Mycobacterium mucogenicum* DSM 44124 | NA | NA | 6099273 | 1 | 6099273 | 98.94 | 1.11 |  | CP062008.1 |
| *Mycobacterium gordonae* X7091 | NA | NA | 7103222 | 1 | 7103222 | 99.86 | 1.26 |  | CP070973.1 |
| *Mycobacterium lentiflavum* ATCC 51985 | NA | NA | 5901783 | 1 | 5901783 | 99.94 | 0.88 |  | CP092423.2 |
| *Mycobacterium europaeum* CSUR P1344 | NA | NA | 6152523 | 13 | 3131389 | 99.61 | 4.39 |  | CTEC00000000 |
| *Mycobacterium numidiamassiliense* AB215 | NA | NA | 6248949 | 4 | 4451009 | 99.61 | 1.97 |  | FUEZ00000000 |
| *Mycobacterium ahvazicum* AFP003 | NA | NA | 6120821 | 9 | 1699181 | 99.28 | 0.62 |  | FXEG00000000 |
| *Mycobacterium genavense* ATCC 51234 | NA | NA | 4936071 | 25 | 465365 | 99.94 | 1.16 |  | JAGZ00000000 |
| *Mycobacterium nebraskense* AKUC2 | NA | NA | 6539988 | 175 | 117537 | 99.94 | 2.32 |  | LDPP00000000 |
| *Mycobacterium paraense* FI-10043 | NA | NA | 5806129 | 51 | 236783 | 99.94 | 0.78 |  | LQPL00000000 |
| *Mycobacterium szulgai* DSM 44166 | NA | NA | 6672659 | 178 | 95710 | 99.61 | 0.56 |  | LQPW00000000 |
| *Mycobacterium triplex* DSM 44626 | NA | NA | 6366285 | 48 | 258006 | 99.57 | 1.03 |  | LQPY00000000 |
| *Mycobacterium paraffinicum* M11 | NA | NA | 6475312 | 85 | 180675 | 99.61 | 1.38 |  | MPNT00000000 |

NA, not applicable

**Supplementary references**

1. Komine T, Srivorakul S, Tomaru K, Tanaka Y, Inohana M, Fukano H, et al. Outbreak of nontuberculous mycobacteriosis with dematiaceous fungus co-infection in aquarium-reared sharphead flyingfish (*Hirundichthys oxycephalus*). Fish Pathol (2021) 56:187–98. doi: [10.3147/jsfp.56.187](https://doi.org/10.3147/jsfp.56.187).

2. Telenti A, Marchesi F, Balz M, Bally F, Böttger EC, Bodmer T. Rapid identification of mycobacteria to the species level by polymerase chain reaction and restriction enzyme analysis. J Clin Microbiol (1993) 31:175–8. doi: [10.1128/jcm.31.2.175-178.1993](https://doi.org/10.1128/jcm.31.2.175-178.1993).

3. Antuofermo E, Pais A, Polinas M, Cubeddu T, Righetti M, Sanna MA, et al. Mycobacteriosis caused by *Mycobacterium marinum* in reared mullets: first evidence from Sardinia (Italy). J Fish Dis (2017) 40:327–37. doi: [10.1111/jfd.12515](https://doi.org/10.1111/jfd.12515).

4. Kumar S, Stecher G, Li M, Knyaz C, Tamura K. MEGA X: Molecular evolutionary genetics analysis across computing platforms. Mol Biol Evol (2018) 35:1547–9. doi: [10.1093/molbev/msy096](https://doi.org/10.1093/molbev/msy096).
