# Supplementary figures and images for: Non-tuberculous mycobacterial disease associated with Mycobacterium montefiorense in salamanders
Source: Front Vet Sci. 2023 Oct 26;10:1248288. doi: 10.3389/fvets.2023.1248288 (PMC10637390; doi:10.3389/fvets.2023.1248288)

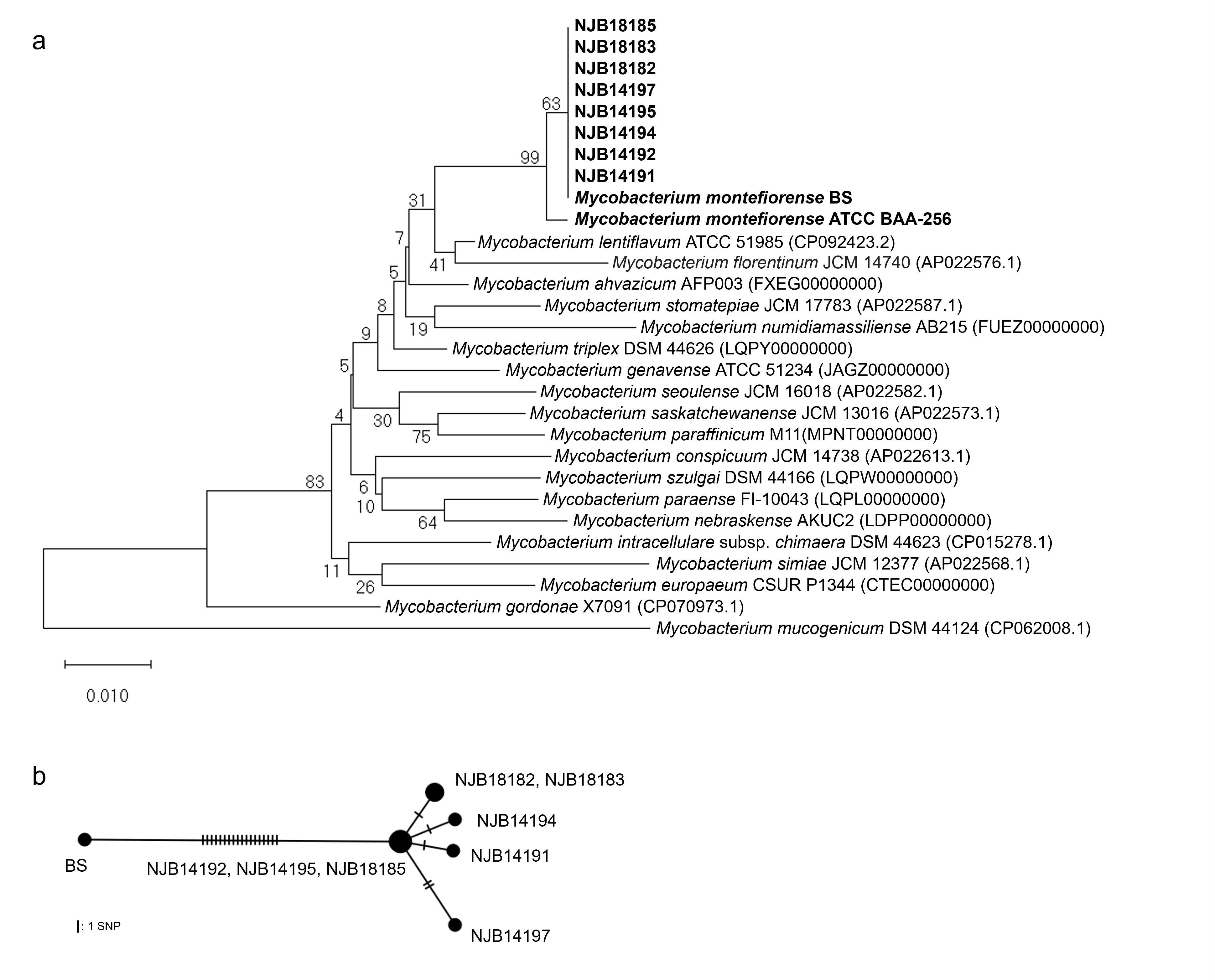

Supplement: Supplementary file 3 [file Image_1.TIF]

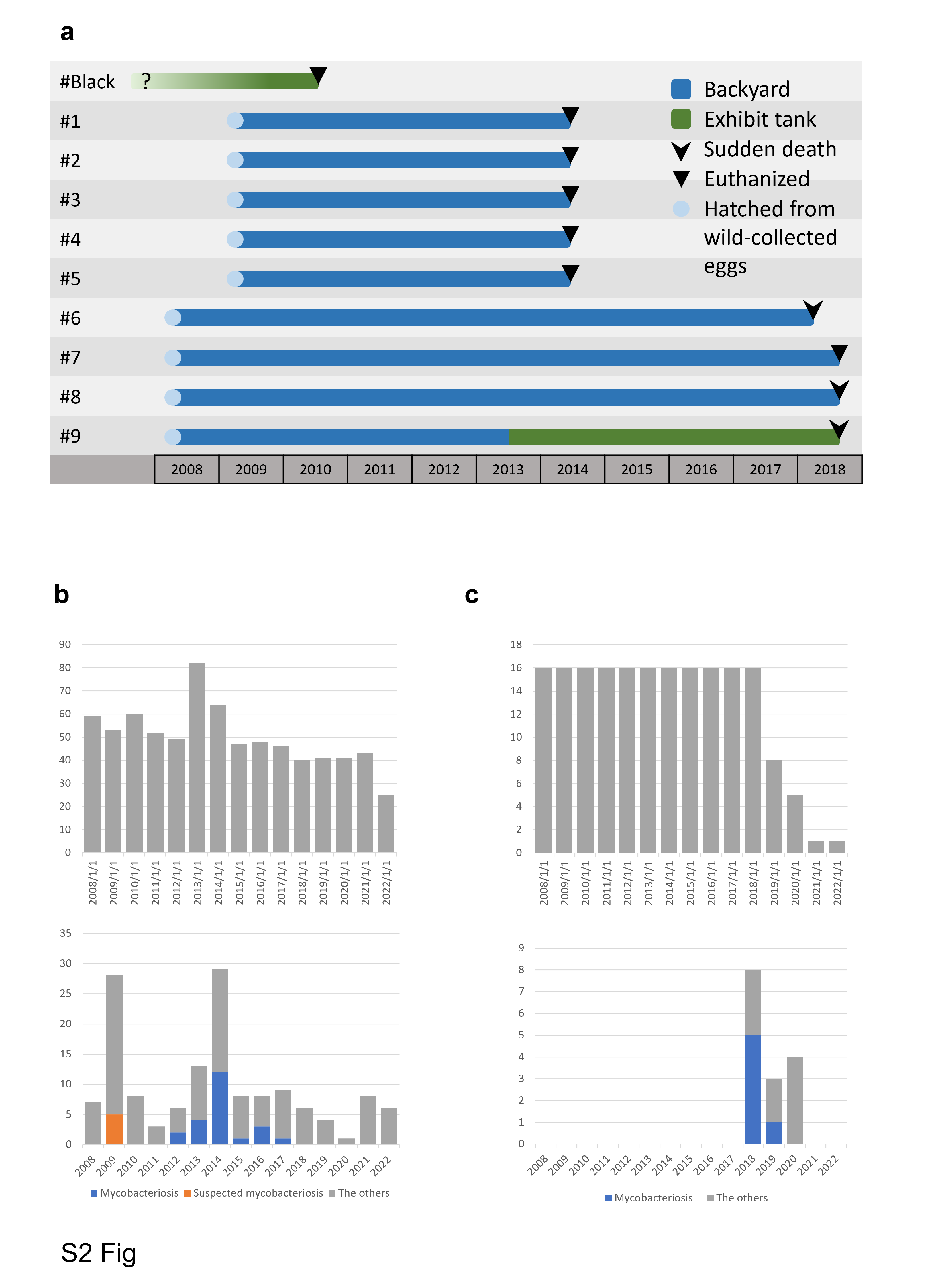

Supplement: Supplementary file 4 [file Image_2.TIF]
